# Guidance Prostheses for Partial Mandibulectomy Patients: A Case Series

**DOI:** 10.7759/cureus.30132

**Published:** 2022-10-10

**Authors:** Vineet Sharma, Jyoti Paliwal, Ashish Dadarwal, Kamal K Meena

**Affiliations:** 1 Prosthodontics, Rajasthan University of Health Sciences (RUHS) College of Dental Sciences, Jaipur, IND

**Keywords:** mandibular guidance therapy, twin occlusal appliance, guidance ramp, guidance prosthesis, mandibulectomy

## Abstract

This case series discusses the fabrication of guidance prostheses (GPs) for patients who presented to the Department of Prosthodontics with marked mandibular deviation, resulting in facial disfigurement and deranged occlusion. These GPs guide the mandible to the unresected side to achieve stable occlusion. This case series included three approaches to reducing mandibular deviation: a maxillary guidance ramp, a mandibular guidance prosthesis, and a twin occlusal appliance. These approaches were used in conjunction with a well-planned mandibular exercise regimen. The earlier mandibular guidance therapy is started, the better the outcome. GPs are used until good occlusal relationships and proprioception are restored. These GPs can be discarded or used occasionally once a good occlusal relationship has been achieved.

## Introduction

Surgical resection of the tumor is associated with secondary disability, significantly when mandibular continuity is not restored. The resection is associated with facial disfigurement, concurrent with the extent of surgery and the reconstruction by soft tissue flaps [1]. There is simultaneous retrusion of the mandibular segment and a deviation of the segment to the surgical site. During mastication, the envelope of function is restricted toward the resected side [2]. As a result, protrusive and laterotrusive (unresected side) movements are restricted. The proprioceptive perception of occlusion and muscle attachment was lost, resulting in uncoordinated, deviated mandibular movements at the surgical site. In addition, the deviation is further aggravated by scar contraction and tight wound closure [2]. Mandibular guidance therapy (MGT) begins when the immediate post-surgical sequelae have subsided, usually about two weeks post-surgery [3]. The earlier MGT is initiated, the more successful the result is. To reduce scar contracture and trismus and improve the maxillomandibular relationship, the patient is instructed to move his mandible away from the surgical site to achieve maximum mouth opening. Notes are made periodically to describe the progress made by the patient [4]. The guidance prostheses (GPs) may be constructed for the mandible or maxilla. GPs are used interim until acceptable occlusal relationships and proper proprioception can be re-established. As soon as an excellent occlusal relationship has been established, the GPs can be discarded or occasionally used to reinforce proprioception [5].

## Case presentation

Three patients with segmental mandibulectomy were rehabilitated with GPs at the Rajasthan University of Health Sciences (RUHS) College of Dental Sciences, Jaipur. The continuity of the mandible was not restored, and all the patients reported lateral and retrusive mandibular deviation toward the resected side. The patients showed a shift in the midline and 2-4 millimeters (mm) of deviation toward the resected side at the maximal mouth opening. In all the cases, the prime objective of the treatment was to achieve appropriate, stable interocclusal contacts. All the patients followed a dedicated exercise program for muscle training followed by maxillary or mandibular GPs.

Case 1

**Figure 1:**
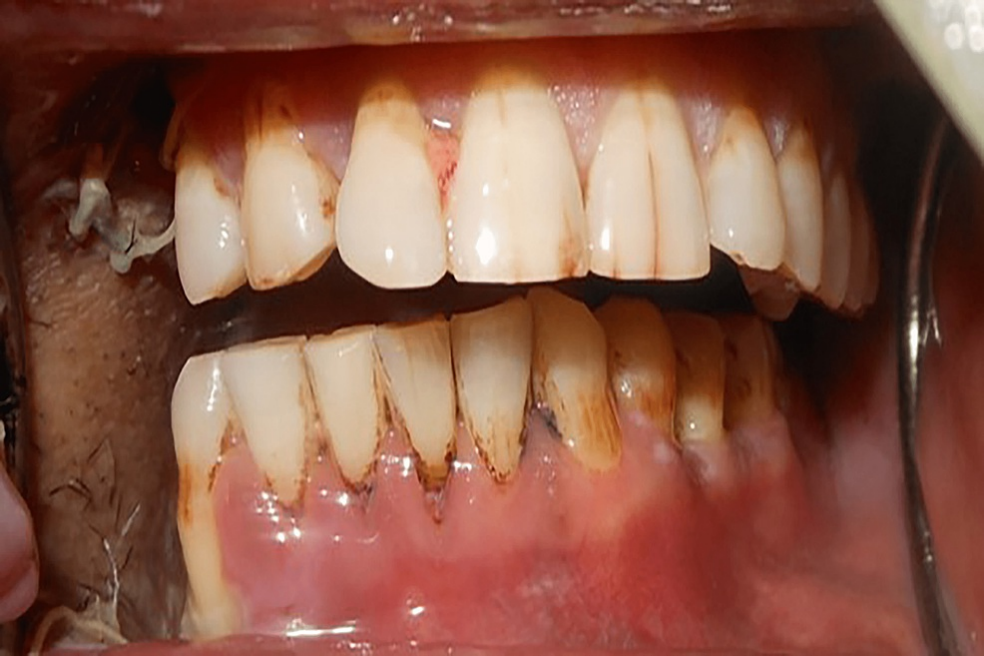
Mandibular deviation during opening

A 52-year-old male presented to the department with the chief complaint of difficulty in chewing because of a facial deformity. The previous history revealed a right-sided mandibulectomy due to oral squamous cell carcinoma (SCC). Extraoral examination revealed a marked deviation of the mandible toward the resected side, which prevented proper contact between the remaining opposing teeth (Figure 1). The defect was identified as a Cantor and Curtis class II defect. As the patient had lost all the molars (45-47) on the right maxillary arch, a guidance ramp (GR) on the maxillary arch was designed to stop the mandibular deviation.

**Figure 2:**
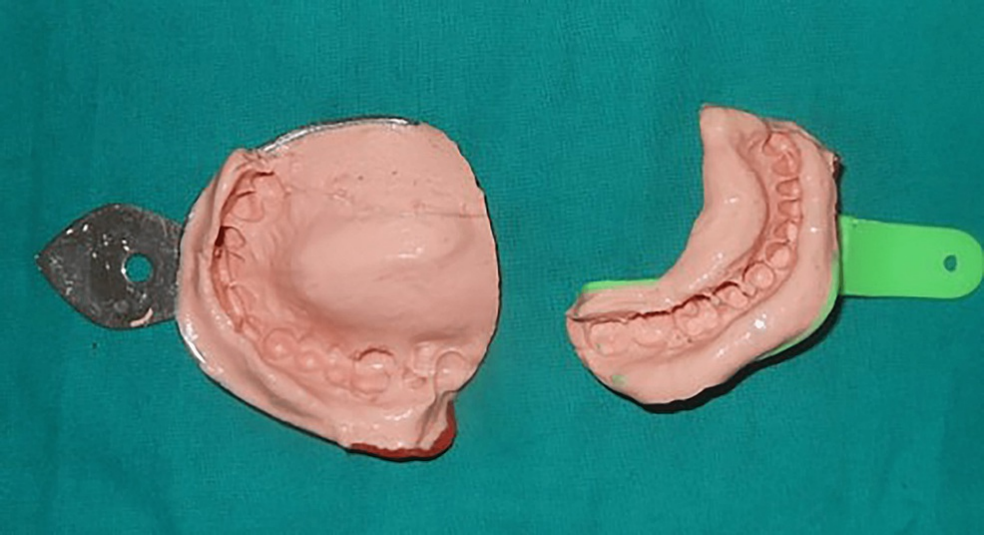
Primary impression using irreversible hydrocolloid impression material

**Figure 3:**
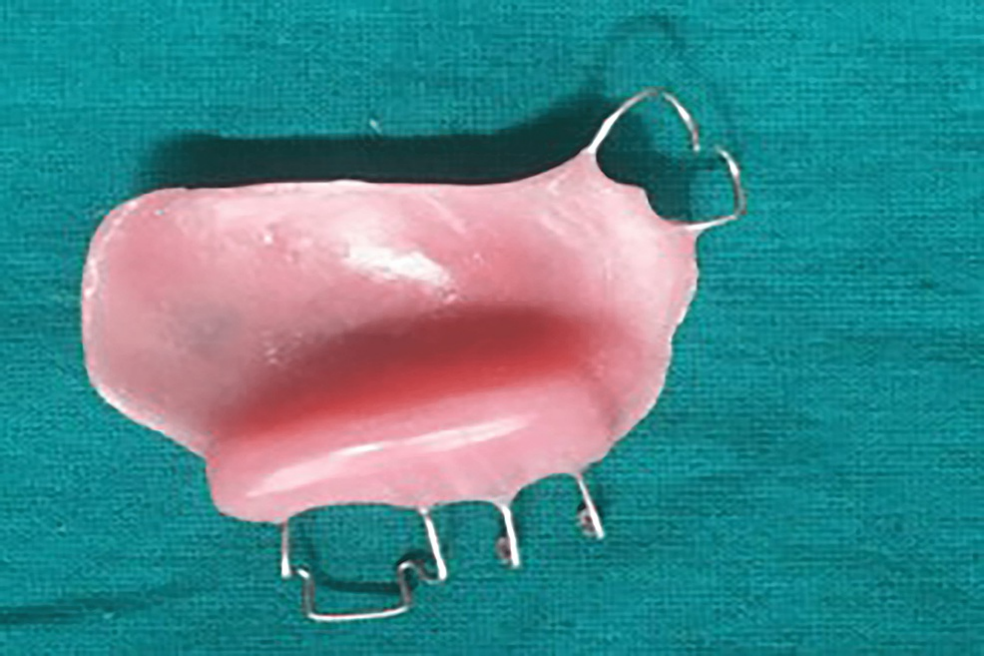
Maxillary guidance ramp

**Figure 4:**
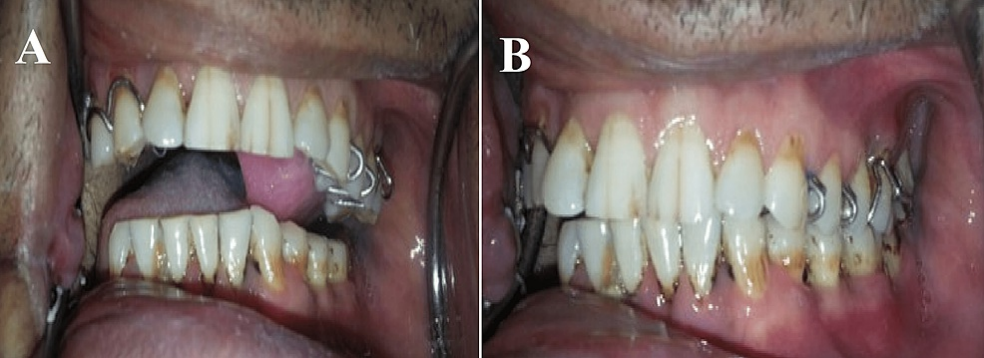
Maxillary guidance ramp appliance in situ. (A) Side view. (B) Achieving proper occlusal relationship.

Primary impressions of both arches were made with irreversible hydrocolloid impression material (Zelgan 2002, Dentsply Sirona, Gurgaon, India) and poured into type III dental stone (Kalstone, Kalabhai Karson, Mumbai, India) (Figure 2). The base plate was fabricated on the maxillary cast with self-cure acrylic resin (ColtoCure C, Coltene, Mumbai, India). A retentive loop was added to the base plate on the unresected side to secure the GR made from hard wax (Modelling Wax Pinnacle Hard; Dentsply Sirona). The mandible was guided toward the unresected side, sliding over the lateral surface of waxed GR until maximum intercuspation on the unresected side was achieved. The maxillary GR was processed with heat-polymerizing acrylic resin (ColtoCure H, Coltene). The maxillary GR was finished and polished after heat processing to reduce mucosal trauma and ensure better tolerability for the prosthesis (Figures 3, 4).

Case 2

**Figure 5:**
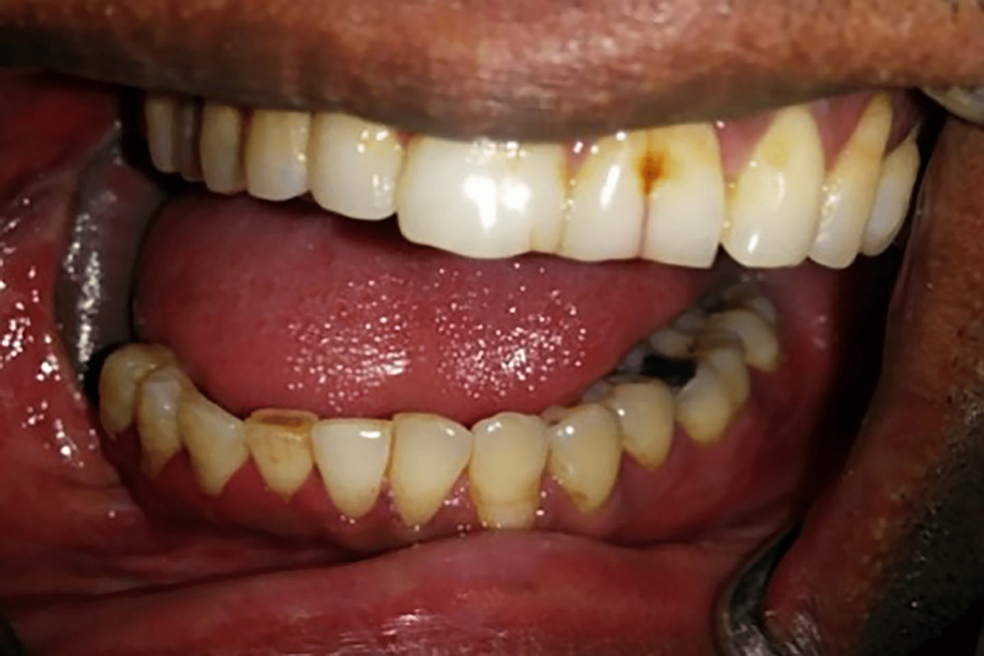
Deviation of the mandible while opening

A 40-year-old male patient was reported to the department with the chief complaint of difficulty in chewing and swallowing after surgery. The history disclosed mandibular SCC followed by right segmental mandibulectomy. A mandibular deviation to the right was observed during an extraoral examination. An intraoral examination revealed missing mandibular right second premolar (45), first molar (46), and second molar (47) (Figure 5). Despite articulating a mediolateral position of the mandible with operator guidance, the patient could not repeat that position consistently enough to achieve proper mastication. The defect was classified as a Cantor and Curtis class II defect. The goal of the treatment was to correct the mandibular deviation. A mandibular guidance prosthesis (MGP) was planned for the case.

**Figure 6:**
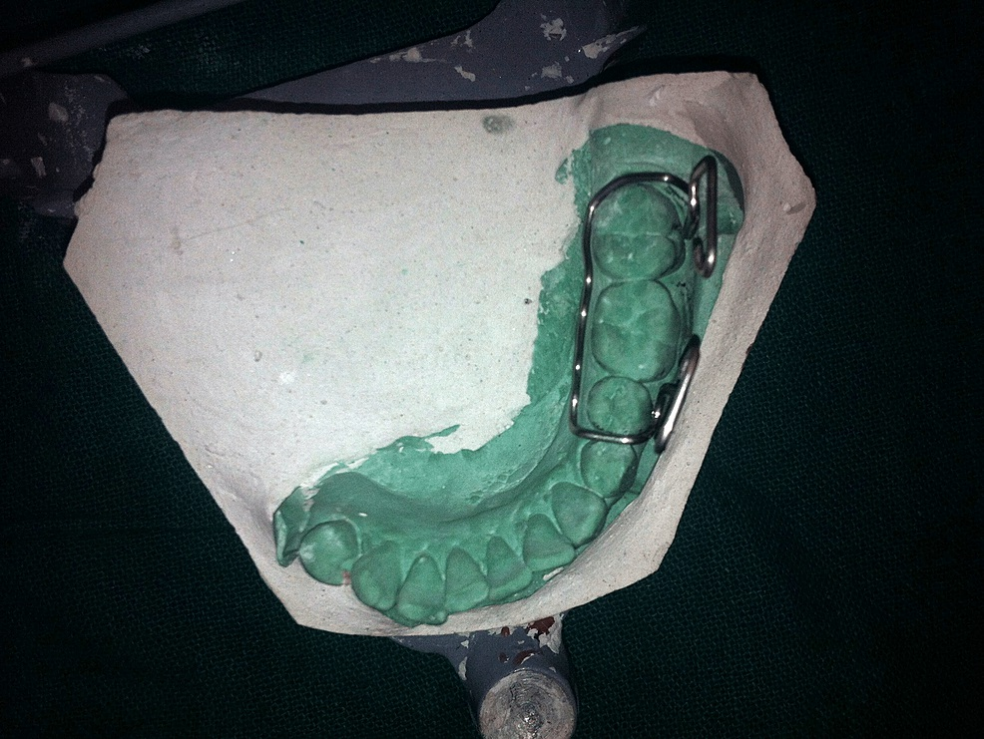
Incorporation of retentive loops

**Figure 7:**
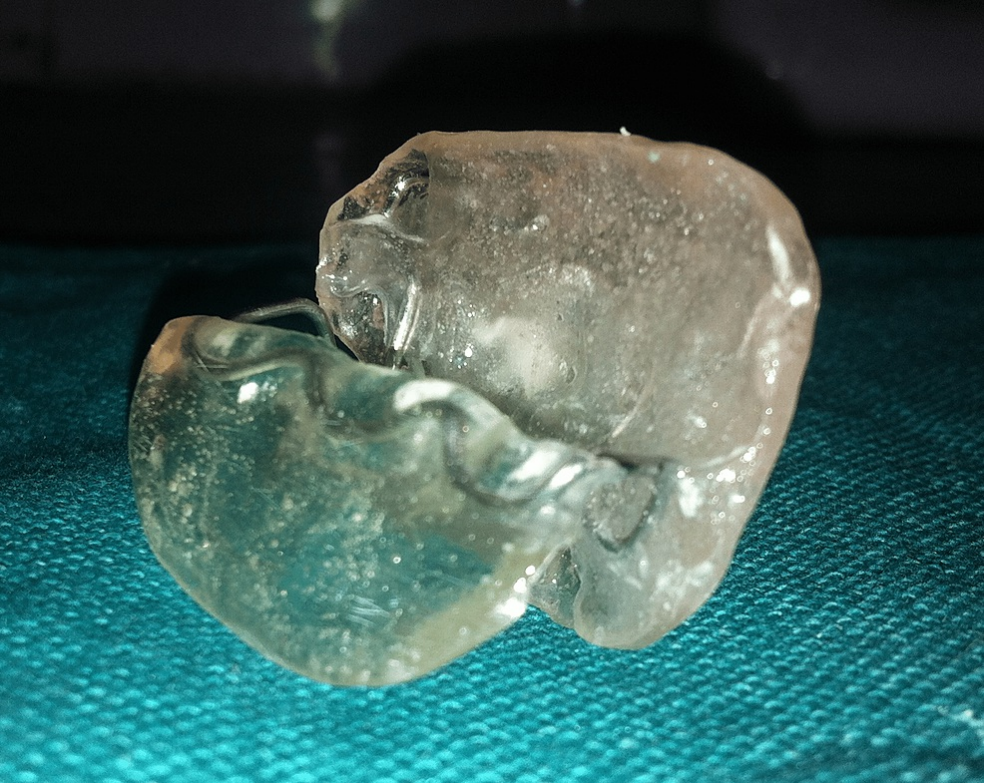
Mandibular guidance prosthesis

**Figure 8:**
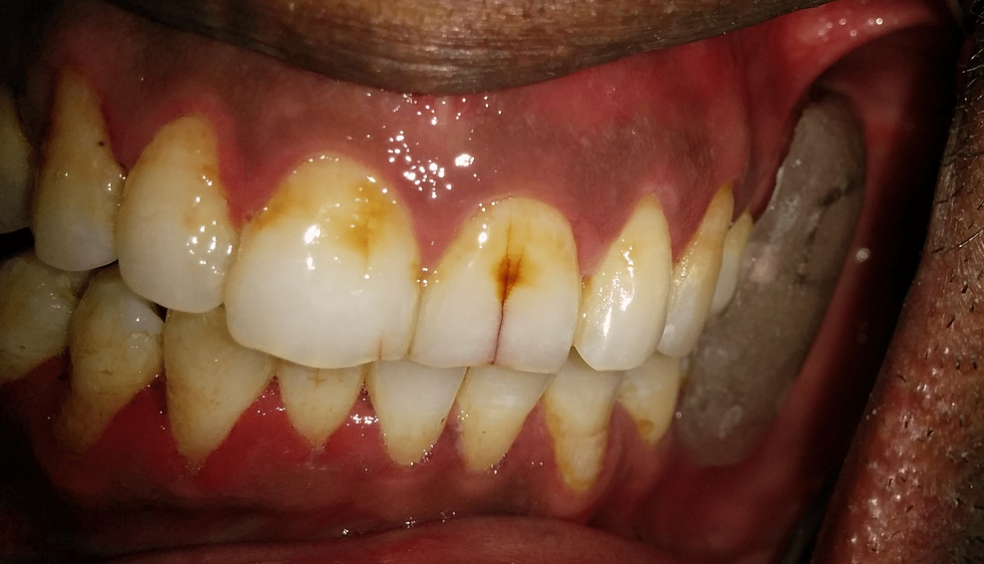
Mandibular guidance prosthesis in situ

Maxillary and mandibular impressions were made with irreversible hydrocolloid impression material (Zelgan 2002, Dentsply Sirona) and poured into a type III dental stone (Kalstone, Kalabhai Karson). A maxillomandibular record was obtained by manually guiding the mandible into the centric occlusion. The maxillary and mandibular casts were mounted on an articulator. The MGP was fabricated on the unresected side. The design incorporated a buccal guidance flange and a lingual supporting flange. Retention was achieved by the interdental clasp, which engaged both premolars and molars. A guide flange (GF) was extended to the buccal surface of each left maxillary molar and premolar, allowing for normal horizontal and vertical overlaps (Figures 6-8). The GF was sufficiently blocked out so that when the patient closed his mouth, it would not traumatize his teeth or gingiva. After finishing the prosthesis, it was evaluated before being inserted intraorally. This GF restricted the movement of the mandible toward the resected site. The appliance was to be worn at all times except when asleep.

Case 3

A 53-year-old patient presented to the department with the chief complaint of difficulty in chewing. The history revealed SCC of the mandible followed by segmental mandibulectomy of the right side. Extraoral examination showed mandibular deviation to the right. The defect was identified as a Cantor and Curtis class II defect. The mandible deviated toward the right side. Despite manually guiding the mandible, occlusal contact between the mandibular and maxillary teeth could not be achieved. Therefore, the maxillary prosthesis included an extra row of teeth for occlusal contacts on the unresected side. As a result, a maxillary twin occlusal appliance (TOA) was made.

**Figure 9:**
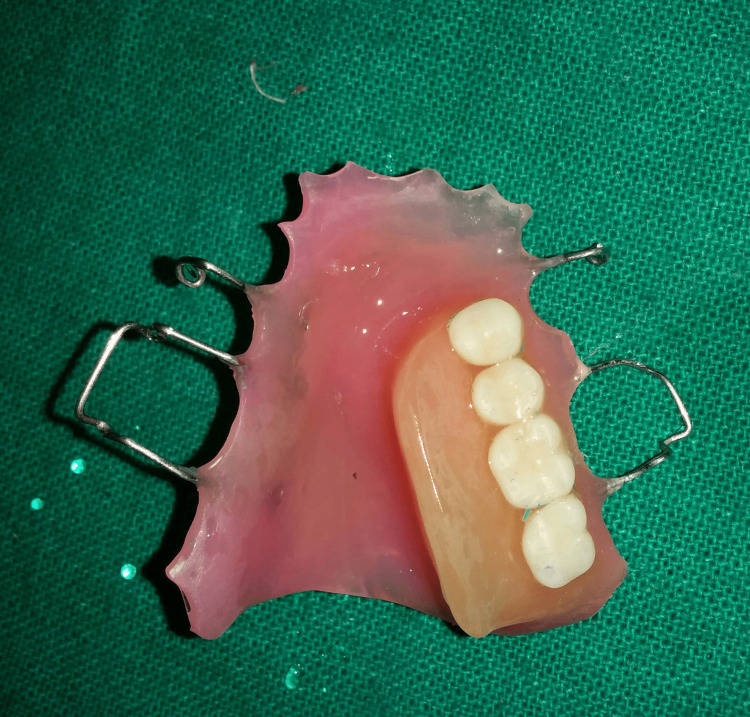
Twin occlusal appliance

**Figure 10:**
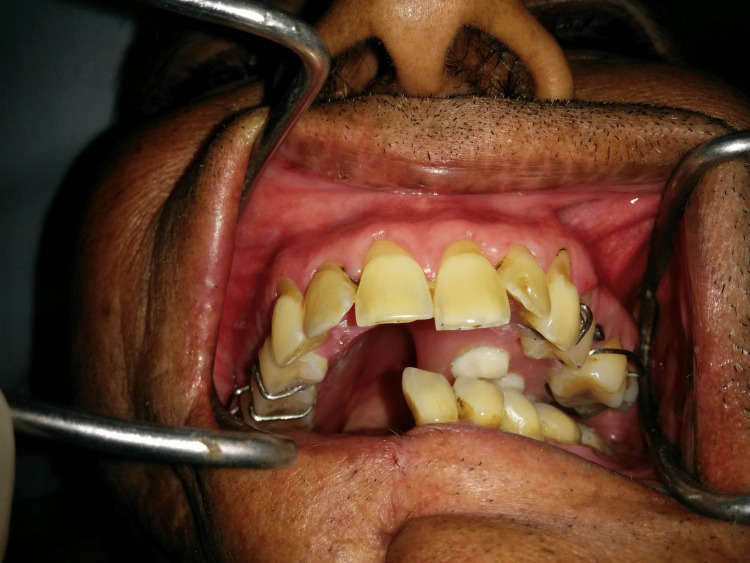
Twin occlusal appliance in situ

Maxillary and mandibular impressions were made with irreversible hydrocolloid impression material (Zelgan 2002, Dentsply Sirona) and poured into a type III dental stone (Kalstone, Kalabhai Karson). The base plate was fabricated on the maxillary cast using self-polymerizing acrylic resin (ColtoCure C, Coltene). Modeling wax was added to the base plate to obtain the functional record. The patients were advised to move the mandible as far as possible onto the unresected side and then gently close the mandible into that position to obtain a functional record. Then, an additional row of teeth was placed in the recorded position. Phonetics and occlusion were evaluated through trial and error. The prostheses were processed with heat-polymerizing acrylic resin (ColtoCure H, Coltene) (Figure 9). The prostheses were finished and polished, and the patients were trained to utilize the secondary occlusal table for mastication (Figure 10).

## Discussion

The loss of mandibular continuity disrupts the mandible's balance and symmetry, resulting in altered movements and deviation of the remaining section toward the surgical site [6,7]. Various complex and interrelated factors such as scar contracture, tight wound closure, and muscle imbalance affect the post-surgical envelope of motion and the deviation severity. Patients who undergo tumor ablation without reconstructive procedures and primary wound closure with radiation therapy usually develop the most severe mandibular deviation and dysfunction [8].

When the resected tissues are replaced with a myocutaneous flap, the severity of the mandibular deviation is minimized, making guidance therapy easier. These findings are based on the premise that exercise is routinely performed shortly after surgery, before, after, and during radiation therapy. Several methods will reduce mandibular deviation, including maxillomandibular fixation (MMF), mandibular or palatal-based guidance restoration, and TOA. However, these methods should be used with a well-planned mandibular exercise plan [9].

MMF was used to reduce the deviation associated with resection of the mandible but is currently not in favor of the immediate placement of arch bars and elastics to achieve MMF. Although rarely used today, this method effectively maintains the proprioceptive sense of the proper intercuspal position during the initial healing period. It should only be used when minimal soft tissue loss is secondary to tumor ablation.

The first design confines the GR and index to a maxillary prosthesis. This form of guidance is indicated for most patients with mandibular deviation. Maxillary GR is more adjustable than MGP and is preferred for patients with severe mandibular deviation [10]. In most instances, these maxillary prostheses are constructed of acrylic resin with cast or wrought wire retainers. They serve only in the interim until an acceptable occlusion can be established. It is recommended that the index does not extend below the level of the maxillary teeth to avoid interfering with speech, deglutition, and other oral functions [11].

When the mandible can be positioned into an acceptable maxillomandibular relationship under operator guidance, but the patient cannot bring the mandible into occlusion, a cast mandibular resection restoration, as recommended by Robinson and Rubright, is used [12]. Restrictive MGP was designed with a flange extending 7 to 10 mm laterally and superiorly on the buccal aspect of the premolars and molars on the non-defect side. This flange engaged the maxillary teeth during mandibular closure, directing the mandible to an optimal intercuspal position [13-15]. The GF can be made of cast chrome-cobalt metal or acrylic resin. The material of choice is determined by the patient's existing occlusal relationship and the need for adjustability. A cast metal MGP will be appropriate if the mandible can be comfortably manipulated into a proper occlusal position. If there is some resistance in the positioning of the mandible, an MGP made of acrylic resin is recommended because it can be adjusted regularly as an improved relationship is obtained. A cast metal MGP allows only minimal adjustment [16].

A stable interocclusal relationship may be restricted even under guidance because of severe scar contracture and fibrosis of the remaining tissues. An alternative treatment approach to the guidance appliance is the TOA, which provides an alternative occlusal table for mastication. This approach increased masticatory efficiency and improved quality of life [17-19].

## Conclusions

Prosthetic management with MGT utilizes a biomechanical system to reduce the defect and deviation, which allows easy placement and maintenance and enhances acceptance of the prosthetic phase. The success of MGT varies depending on the type of surgical defect, the timing of the beginning of guidance therapy, patient cooperation, and other variables. MGT is most effective in patients whose resection only involves bony structures with minimal sacrifice of the tongue, the floor of the mouth, and adjacent soft tissues. Patients who have not been irradiated have a better chance of achieving a functional interocclusal position using MGT. This conservative approach promotes a more favorable psychological and physiological equilibrium. As a result, such an intermediate training device assists in directing the mandible to maximal occlusal contacts, hence increasing life quality.
